# Reconstruction and Characterization of Full-Length Begomovirus and Alphasatellite Genomes Infecting Pepper through Metagenomics

**DOI:** 10.3390/v12020202

**Published:** 2020-02-11

**Authors:** Verónica A. Bornancini, José M. Irazoqui, Ceferino R. Flores, Carlos G. Vaghi Medina, Ariel F. Amadio, Paola M. López Lambertini

**Affiliations:** 1 Instituto de Patología Vegetal, IPAVE-CIAP-INTA, 5000 Córdoba, Argentina; verobornancini@gmail.com (V.A.B.); gastonvaghi@gmail.com (C.G.V.M.); 2 Consejo Nacional de Investigaciones Científicas y Técnicas (CONICET), Argentina; irazoqui.matias@gmail.com (J.M.I.); arielfamadio@gmail.com (A.F.A.); 3 Estación Experimental Agropecuaria Rafaela-INTA, 2300 Rafaela, Santa Fe, Argentina; 4 Estación Experimental Agropecuaria Yuto-INTA, 4518 Yuto, Argentina; cefeflores@gmail.com

**Keywords:** *Geminiviridae*, *Geminialphasatellitinae*, RCA-NGS, pepper, recombination

## Abstract

In northwestern Argentina (NWA), pepper crops are threatened by the emergence of begomoviruses due to the spread of its vector, *Bemisia tabaci* (Gennadius). The genus *Begomovirus* includes pathogens that can have a monopartite or bipartite genome and are occasionally associated with sub-viral particles called satellites. This study characterized the diversity of begomovirus and alphasatellite species infecting pepper in NWA using a metagenomic approach. Using RCA-NGS (rolling circle amplification-next generation sequencing), 19 full-length begomovirus genomes (DNA-A and DNA-B) and one alphasatellite were assembled. This ecogenomic approach revealed six begomoviruses in single infections: soybean blistering mosaic virus (SbBMV), tomato yellow spot virus (ToYSV), tomato yellow vein streak virus (ToYVSV), tomato dwarf leaf virus (ToDfLV), sida golden mosaic Brazil virus (SiGMBRV), and a new proposed species, named pepper blistering leaf virus (PepBLV). SbBMV was the most frequently detected species, followed by ToYSV. Moreover, a new alphasatellite associated with ToYSV, named tomato yellow spot alphasatellite 2 (ToYSA-2), was reported for the first time in Argentina. For the Americas, this was the first report of an alphasatellite found in a crop (pepper) and in a weed (*Leonurus japonicus*). We also detected intra-species and inter-species recombination.

## 1. Introduction

*Begomovirus*, the largest genus within the family *Geminiviridae*, comprises viruses with circular ssDNA genome that infect both dicotyledon and monocotyledon plants and are transmitted by the whitefly *Bemisia tabaci* Gennadius (Hemiptera: Aleyrodidae) [1]. Most of the new world (NW) native begomoviruses have bipartite genomes, known as DNA-A and DNA-B (each component being between 2.6 kb and 2.8 kb in size), but there are some with monopartite genome [2,3].

Northwestern Argentina (NWA) is one of the major horticultural production areas where begomoviruses cause economic losses. In this region, sweet pepper (*Capsicum annuum* L.) is an important crop mostly grown under greenhouse conditions with a production area of about 6662 ha [4]. In the Americas, the genetic diversity of begomoviruses identified in pepper is less than that reported in tomato [5]. There is no information about begomoviruses infecting pepper in Argentina. The first records of begomoviruses affecting pepper crops were reported for the United States and Mexico in the 1990s [6,7]. Pepper golden mosaic virus (PepGMV) is widely distributed in Mexico and Central America [8,9,10,11]. PepGMV and pepper huasteco yellow vein virus (PHYVV) are commonly found in mixed infections [12,13]. Furthermore, potato yellow mosaic virus (PYMV) was identified in Trinidad and Tobago, and a squash yellow mild mottle virus (SYMMoV) was reported in Costa Rica [14,15]. In Brazil, the major reports of begomovirus infecting pepper correspond to tomato severe rugose virus (ToSRV) and tomato yellow vein streak virus (ToYVSV) [16,17]. Moreover, the pepper leafroll virus (PepLRV) has been recently reported in Peru [18].

Viral metagenomics (viromics) is a powerful tool for viral diversity exploration in a wide range of environments [19]. When a metagenomic study is linked to a host, it is called ecogenomics [20]. In particular, rolling circle amplification (RCA) [19] as a viral genome enrichment technique, coupled with next-generation sequencing (NGS), has been used to identify geminiviruses, especially begomoviruses and their satellites in different crops [21,22,23,24,25].

Circomics, the combination of RCA-restriction fragment length polymorphism (RCA-RFLP) and pyro-sequencing, has shed light on begomovirus species identification [26,27]. Another similar methodology is vector-enabled metagenomic (VEM), which surveys begomovirus using whiteflies [28,29,30]. VEM involves the purification of virus particles obtained from the insect vector and metagenomic sequencing. Deep sequencing of virion-associated nucleic acids (VANA), small-interfering RNAs (siRNA), and total RNA libraries were also applied for geminivirus identification [31,32,33,34,35]. The application of deep sequencing strategies in virology offers the opportunity to detect mutations, as well as to understand evolutionary strategies and viral population dynamics [36].

This study reported the use of a viral metagenomic approach to the characterization of the diversity of begomovirus and alphasatellite species infecting pepper in NWA. The described protocol allowed the generation of full-length genomic sequences of begomoviruses for their application in phylogenetic and recombination analyses.

## 2. Materials and Methods 

### 2.1. Sampling and RCA-Seq

A total of 101 pepper plants with characteristic symptoms of virus infection were sampled in Pichanal and Oran (Salta province) and Yuto (Jujuy province) from 2005 to 2015. Total DNA from infected apical leaves was purified using Nucleo-Spin Plant II Kit (Macherey-Nagel, Düren, DEU). Begomovirus infection was confirmed by multiplex-PCR with universal degenerate primers targeting DNA-A and RubiscoL primers as a control [18,37]. Positive PCR samples (52) were subjected to RCA to amplify circular DNA using Templi-phi^TM^ (GE Healthcare, Buckinghamshire, UK). RFLP patterns were obtained with ApaI, BamHI, PstI, and XhoI enzymes and evaluated by electrophoresis in a 1.2% agarose gel. Based on the diversity of RCA-RFLP fragments patterns, 20 begomovirus-infected samples were selected for NGS analysis. The selection covered all the observed patterns in an attempt to capture most of the variability among samples. RCA products were purified with Wizard^®^ SV Gel and PCR Clean-Up System (Promega, Madison, USA). Nextera XT DNA libraries were constructed for RCA products and were sequenced (2× 150 bp paired ends) on Illumina HiSeq 1500 system (INDEAR, Rosario, Santa Fe, ARG). 

### 2.2. NGS Data Pipeline

Pre-processing NGS reads generated from RCA (RCA-NGS) consisted of removing adapter and poor-quality sequences using Trimmomatic [38]; quality was assessed by FASTQC [39]. The pre-processed Illumina reads from each sample were de novo assembled using IVA (Iterative Virus Assembler) [40]. The results of each assembly were compared against the NCBI *nt* database using BLAST. For samples that did not produce full-length viral contigs, reads were mapped against the pepper genome (*C. annuum* Zunla Ref_v1.0; Accession number: GCF_000710875.1) using Bowtie2 [41], and mapped reads were removed. The remaining reads were re-assembled, as previously described. Lastly, if the assembly did not result in a complete genome, a reference guided assembly was performed using the closest virus found by BLAST as reference. All contigs were manually inspected to remove RCA repeated extremes using Dotter [42], and then open reading frames (ORF) integrity was checked using Artemis [43]. Pairwise comparisons of DNA-A and DNA-B assembled sequences were first detected against nr database using BLASTN [44], and then pairwise identities were calculated using SDT v1.2 (Species Demarcation Tool) as a begomovirus taxonomy requirement [45]. Moreover, the intra-species identity of *Soybean blistering mosaic virus* (SbBMV) and *Tomato yellow spot virus* (ToYSV) was compared using SDT v1.2.

### 2.3. Cloning and Sanger Sequencing of the New Begomovirus Species Identified by RCA-NGS

In order to validate the de novo-assembled sequences obtained by RCA-NGS from pepper sample 663, the genomic components were cloned and Sanger-sequenced for their comparison. RCA products were digested with XhoI and KpnI to release DNA-A and DNA-B, respectively. Monomers were obtained by agarose gel purification, ligated into a digested dephosphorylated pBluescript SK+ vector (Stratagene, La Jolla, CA) and transformed into *Escherichia coli* JM109. Plasmids carrying DNA-A or DNA-B were sequenced in both orientations using a primer walking strategy (Macrogen Inc., Seoul, South Korea). DNA-A and DNA-B sequences were assembled with Geneious v9.1.5 [46]. Begomovirus full-length genome sequences, obtained by RCA-NGS and Sanger sequencing, were compared by pairwise genetic identity calculation using SDT v1.2.

### 2.4. Cloning and Sanger Sequencing of ToYSV and a New Associated Alphasatellite from a Weed Sample

It was not possible to assemble a full-length DNA-A of begomovirus by RCA-NGS from pepper sample 423, nor was it possible to clone one. Therefore, weed sample 417 (*Leonurus japonicus* Houtt), which was taken from the same field as pepper sample 423 and showed the same RCA-RFLP patterns as those of BamHI (2.6 kb and 1.4 kb fragments), was selected for cloning, sequencing, and comparison.

RCA products from weed sample 417 were digested with ApaI to release DNA-A, and with BamHI to release DNA-B and the alphasatellite genome. Monomers were gel-purified, ligated into a digested and dephosphorylated pBluescript SK+ vector (Stratagene, La Jolla, CA), and transformed into *E. coli* JM109. Plasmids carrying the full genomic components were sequenced by primer walking (Macrogen Inc., Seoul, South Korea). DNA-A, DNA-B, and alphasatellite sequences were assembled with Geneious v9.1.5, and the nucleotide identity was calculated by SDT v1.2.

### 2.5. Phylogenetic Analysis of Assembled Begomovirus Sequences

The phylogenetic relationships of full-length assembled sequences generated in this work were analyzed with all South American begomovirus sequences reported in GenBank (December 2018). A set of 100 DNA-A sequences and 81 DNA-B sequences were aligned using the MUSCLE program implemented in Geneious v9.1.5. Maximum likelihood (ML) phylogenies for both DNA-A and DNA-B were inferred using RAxML v8.2 [47], using the GTR GAMMA+I model and 1000 bootstraps. The resulting trees were plotted using iTOL v5 (Interactive Tree Of Life) [48].

### 2.6. Phylogenetic Analysis of Alphasatellite Sequences

Phylogenetic relationships of alphasatellite sequences obtained from pepper sample 423 and weed sample 417 were analyzed with worldwide geminivirus-associated alphasatellite sequences reported in GenBank (at date 10-01-2018). A set of 46 alphasatellite sequences were aligned in Geneious v9.1.5 by applying the MUSCLE algorithm and the phylogenetic tree using RAxML v8.2, with the GTR GAMMA+I model and 1000 bootstraps. The resulting tree was plotted using iTOL.

### 2.7. Recombination Analysis

Potential recombination sequences were detected with RDP4 v4.95 (Recombination Detection Program) using RDP, GENECONV, MaxChi, Bootscan, 3Seq, Chimaera, and Siscan methods implemented with default setting [49]. The DNA-A and DNA-B alignments obtained for phylogenetic analysis were filtered at a 69% sequence identity. Only breakpoints with Bonferroni-corrected *p* < 0.05 were reported. The complex patterns of recombination characterized using RDP4 were manually checked, taking into account the following different characteristics: event number, support in different phylogenetic trees, determination of breakpoint position with different methods and matrix-based visualizations, and statistical and phylogenetic tests.

In addition, an intra-species recombination analysis for SbBMV and ToYSV was performed by RDP4 and phylogenetic network using SplitsTree4 v4.14.6 [50]. First, DNA-A sequences of SbBMV or ToYSV generated in this work and previously reported were aligned with MUSCLE. The presence of significant recombination was tested using a phylogenetic network along with the PHI test (pairwise homoplasy index test) estimated with *p* < 0.05 [51]. To detect recombinant sequences, each sequence was progressively removed from alignment until the PHI test for the remaining sequences was no longer significant (*p* > 0.05) [52].

## 3. Results

### 3.1. Reconstruction of Begomovirus and Alphasatellite Genomes Infecting Pepper

Begomovirus infections were detected by multiplex-PCR in 51.48% of the pepper samples collected in NWA. RCA-NGS analysis was done in 20 of those samples (Table 1). After trimming, all samples retained at least 1.6 million reads that were then assembled. IVA assembly resulted in a small number of contigs, which were manually inspected. Most of them corresponded to viral contigs, and the remaining ones were easily identified as pepper fragments. We analyzed the fraction of reads corresponding to the pepper genome and found it to be variable, between 12% and 87% (Table 1). 

For 20 samples, 19 DNA-A, 20 DNA-B, and one alphasatellite full-length genome sequences were reconstructed (Table 2). For sample 423, no DNA-A was obtained, but a DNA-B and an alphasatellite were reconstructed. On average, the pepper genome accounted for over 59% of each sample, but for most cases, this was not an obstacle to recover the full-length viral genome. The elimination of mapped reads of the pepper genome was necessary to improve the assembly results in only five samples (302, 423, 588, 654, and 663). The assembly of the filtered reads from sample 654 resulted in one contig, containing both DNA-A and DNA-B. Hence, a reference assembly strategy was chosen, using the SbBMV as a template, leading to complete DNA-A and DNA-B for this sample. For sample 302, three small contigs were obtained after eliminating pepper reads, all matching with sida golden mosaic Brazil virus (SiGMBRV, also mentioned Sida Brazil virus in Genbank). Using this virus as a template, both full-length DNA-A and DNA-B were reconstructed.

Different validation steps of sequences obtained by RCA-NGS were performed. One consisted of comparing the RFLP patterns obtained in silico from the generated sequences with those obtained by RCA-RFLP of the same samples. The patterns achieved were similar for each pepper sample, validating the results of this methodology (Table S1). Likewise, new begomovirus and alphasatellite species were validated by comparison with sequences obtained by Sanger (Table 3). This validation step was made to discard possible chimeric sequences generated by a wrong assembly result since begomoviruses are usually in mixed infections and share a high percentage of nucleotide identity.

**Figure 1:**
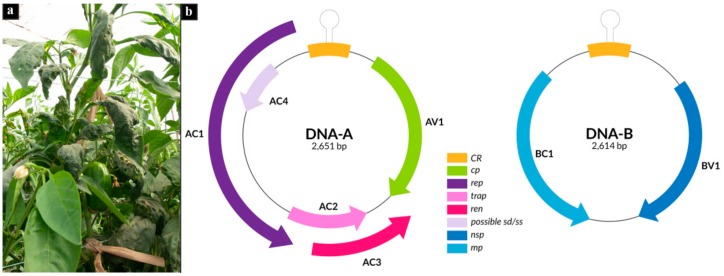
(**a**) Symptoms of blistering, curling, and chlorosis of pepper leaves infected with pepper blistering leaf virus (PepBLV). (**b**) Genome organization of PepBLV. Open reading frames (ORFs) (AV1, AC1, AC2, AC3, AC4, BV1, BC1) are color-coded according to the possible function of their protein products (*cp*, capsid protein; *rep*, replication-associated protein; *trap*, transactivator protein; *ren*, replication enhancer; *sd*, possible symptom determinant; *ss*, possible silencing suppressor; *nsp*, nuclear shuttle protein; *mp*, movement protein). CR, common region, with the hairpin, is indicated.

This ecogenomic approach revealed single begomovirus infection by six begomovirus species infecting pepper in Argentina: SbBMV, ToYSV, ToYVSV, tomato dwarf leaf virus (ToDfLV), SiGMBRV and a newly proposed species of begomovirus (Table 2). In addition, a new alphasatellite infecting pepper was identified (sample 423, Table 2). SbBMV was the most abundant species in the selected samples, followed by ToYSV (Table 2). DNA-A de novo assembled from pepper sample 663 shared 83.1% of sequence identity with the solanum mosaic Bolivia virus (SoMBoV, HM585435) (Table 2). Since the nucleotide identity was below 91%, the corresponding threshold for species demarcation [53], we proposed this as a new begomovirus species, with the name pepper blistering leaf virus (PepBLV), according to symptoms observed in pepper field plant (Figure 1a). DNA-B de novo assembled from pepper sample 663 shared 83.7% of sequence identity with the DNA-B of sida mosaic Bolivia virus-1 (SiMBoV1, NC015044). PepBLV DNA-A was 2651 bp in size and contained six ORFs: AV1, AC1, AC2, AC3, and AC4. DNA-B was 2614 nt in size with two ORFs: BV1 and BC1, according to the bipartite genome organization of new world begomoviruses (Figure 1b). The common region between DNA-A and DNA-B shared 98% identity. The comparison between the sequences of PepBLV DNA-A and DNA-B obtained by RCA-NGS (Table 2) and those obtained by Sanger sequencing (Table 3) showed at least 99.84% nucleotide identity. Thus, the RCA-NGS and de novo assembly procedures showed to be a valid approach for the identification and molecular characterization of new begomovirus species.

The results for pepper sample 423 were challenging because only one full-length DNA-B and one alphasatellite sequence were obtained by de novo assembly procedure. No DNA-A full-length sequences were obtained by RCA-NGS or cloning. The DNA-B sequence showed 95.9% nucleotide identity with ToYSV DNA-B (KJ742420) (Table 2). The shorter sequence obtained by de novo assembly showed 85.1% identity with tomato yellow spot alphasatellite (KX348228) (Table 2); therefore, it should be classified as a new species according to the 88% threshold demarcation criteria for the recently established subfamily *Geminialphasatellitinae* in the family *Alphasatellitidae* [54]. With the purpose of confirming the begomovirus species that is associated with this new alphasatellite, weed sample 417 (*Leonurus japonicus* Houtt), which showed the same RCA-RFLP pattern with BamHI (2.6 kb and 1.4 kb fragments) as the pepper sample 423, was selected for cloning and Sanger sequencing. Full-length sequences of DNA-A (2632 bp), DNA-B (2595 bp), and two alphasatellite sequences (1350 bp) were obtained from this sample. DNA-A sequence (MN518741) displayed 98.9% of nucleotide identity with ToYSV DNA-A (KJ742419) (Table 3). DNA-B (MN518741) presented 96% of nucleotide identity with ToYSV DNA-B (KJ742420). The two alphasatellite sequences (MN518743 and MN518744) showed the same nucleotide identity (84.2%) with tomato yellow spot alphasatellite (KX348228) (Table 3). The nucleotide identity between alphasatellite sequences from pepper sample 423 (MN518745, obtained by NGS) and weed sample 417 (MN518743 and MN518744, obtained by Sanger) was 98.2%; therefore, all the sequences belonged to this new proposed alphasatellite. Again, the comparison of sequences obtained by both methods (RCA-NGS and Sanger-sequencing of clones) demonstrated the accuracy of this ecogenomic approach. Finally, the name tomato yellow spot alphasatellite 2 (ToYSA 2) was proposed for this putative new alphasatellite because it was found to be associated with ToYSV in *C. annum* and *L. japonicus.* This alphasatellite presented one open reading frame coding for a potential replication-associated protein (*rep*) and the conserved hairpin structure, the organization predicted for *Geminialphasatellitinae* species.

**Figure 2:**
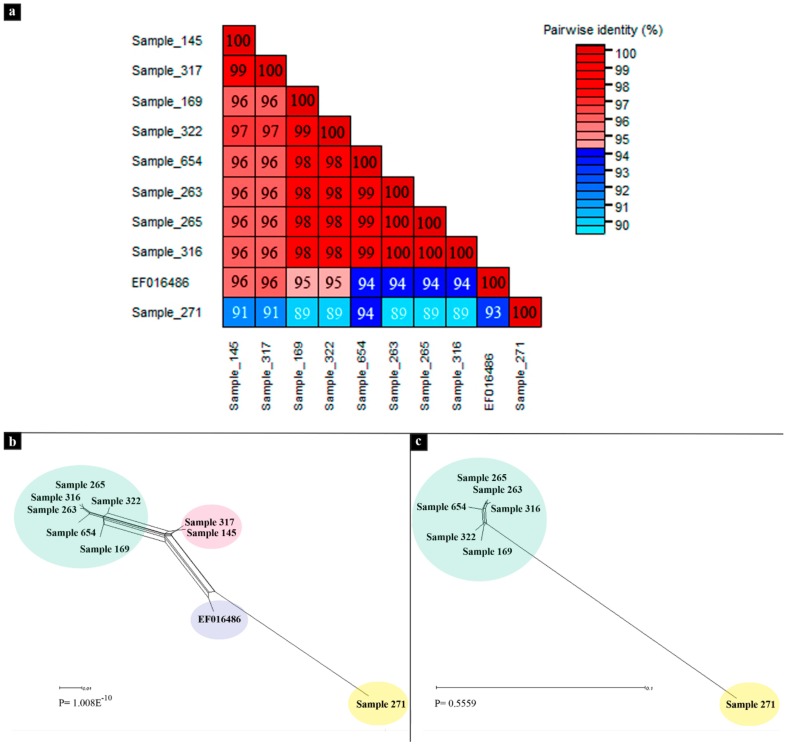
(**a**) Pairwise identity matrix of all soybean blistering mosaic virus (SbBMV) DNA-A sequences inferred using Species Demarcation Tool (SDT) v1.2. (**b**) Phylogenetic networks for all SbBMV DNA-A sequences obtained with the NNet algorithm using Splits Tree. Pairwise homoplasy index (PHI) test indicated evidence of recombination between the different SbBMV isolates; (**c**) Phylogenetic networks for SbBMV DNA-A sequences, excluding the recombinant sequences (Sample 145, Sample 317, and EF016486) and nonsignificant PHI test.

**Figure 3:**
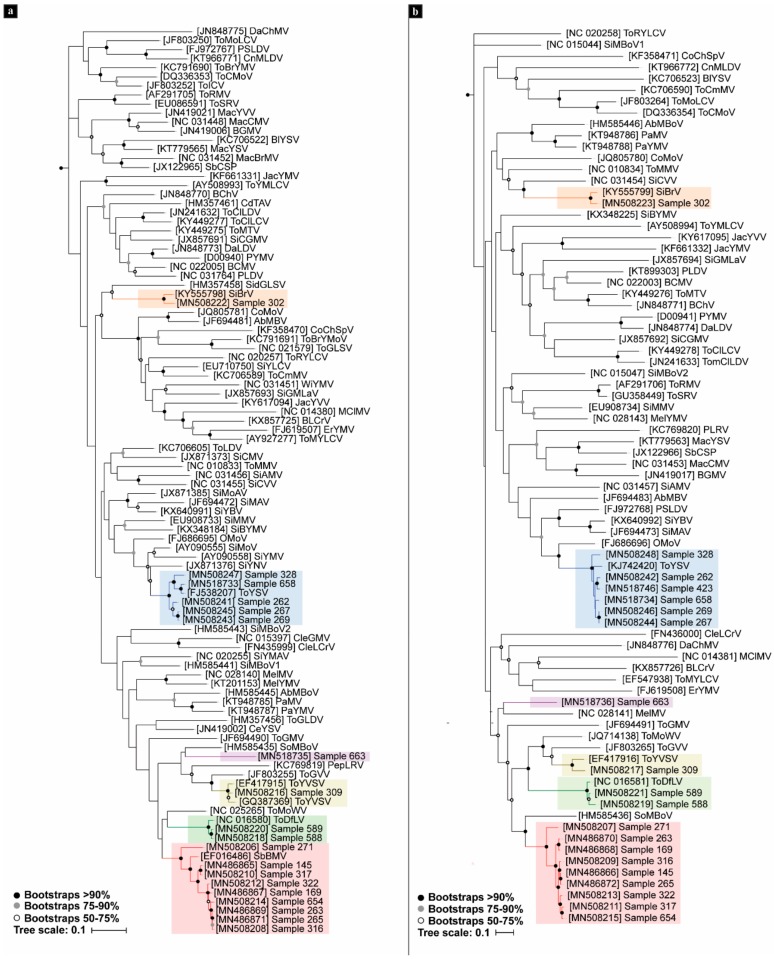
Maximum likelihood phylogenetic tree of full genome sequences of begomoviruses infecting pepper in northwestern Argentina (NWA) and the representative South American begomoviruses. Branches with more than 50% bootstrap support are shown: (**a**) DNA-A tree rooted with tomato leaf curl New Delhi virus DNA-A (U15015); (**b**) DNA-B tree rooted with tomato leaf curl New Delhi virus DNA-B (U15017).

Regarding SbBMV, the pairwise nucleotide sequence comparison between the nine SbBMV sequences generated in this work with the one previously reported in Genbank was an example of conflict-resolution criteria for strain begomovirus taxonomy (Figure 2a) [53]. SbBMV (Sample 271) shared the highest percent identity (94%) with isolate (Sample 654) and ≤94% with all other SbBMV isolates; therefore, it was not considered a new strain, although, in the phylogenetic analysis, it was distantly related to the other isolates (Figure 2a,b, and Figure 3).

Likewise, the pairwise sequence comparisons between all ToYSV sequences (five generated in this work and 21 reported) did not allow us to identify virus strains with the current taxonomy criteria (Figure S1a).

The sequences of SbBMV DNA-A from NWA (nine isolated from pepper and one from soybean) presented an overall nucleotide sequence identity that varied from 88.9% to 99.7%, resulting in the highest degree of genetic variability detected (Figure 2a). For ToYSV DNA-A (five isolated from pepper, one from *Leonurus,* and two previously reported isolated from bean and chia), the nucleotide sequence variability was lower, from 94.7% to 98.5% identity (Figure S1a, Table 3). The genetic variability of DNA-B sequences was similar for both viruses, showing nucleotide sequence identity from 96.5% to 99.8% for SbBMV and from 94.5% to 97.4% for ToYSV.

### 3.2. Phylogenetic Analyses

A phylogenetic tree based on the complete DNA-A sequence of the begomoviruses from pepper and other reported South American begomoviruses was constructed (Figure 3a). The pepper-infecting begomoviruses were placed in three different clusters: one including SbBMV, ToYVSV, ToDfLV, and PepBLV; another one containing ToYSV with Brazilian begomovirus from weeds; and the other including SiGMBRV. PepBLV (the proposed new species) showed a closer phylogenetic relationship with SoMBoV (HM585435) (Figure 3a). PepBLV DNA-B showed a closer phylogenetic relationship with MelMV (NC-028141) (Figure 3b). Interestingly, although the DNA-B sequences retained their location in three different groups, such as those in DNA-A, the closest relationships were not the same as those observed in the tree for the DNA-A sequence (Figure 3a,b).

**Figure 4:**
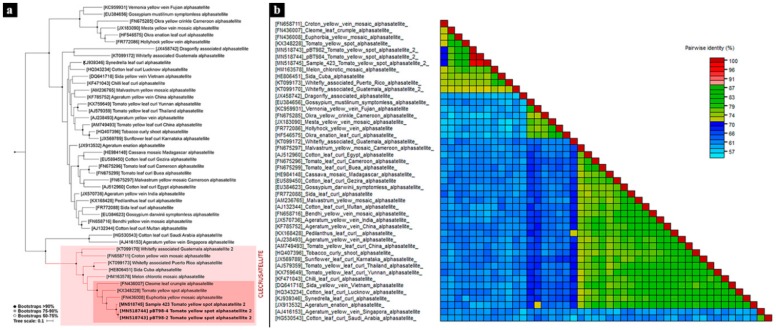
(**a**) Maximum likelihood phylogenetic tree of tomato yellow spot alphasatellite 2 (ToYSA-2) and the representative geminivirus-associated alphasatellite sequences reported up to now and rooted with a representative group of DNA-R sequence of nanoviruses. Branches with less than 50% bootstrap support have been collapsed; (**b**) A “three color” pairwise identity matrix of alphasatellite data set inferred using SDT v1.2.

The phylogenetic tree of all complete alphasatellite sequences showed that the proposed new species, ToYSA 2, shared a common ancestor with euphorbia yellow mosaic alphasatellite (FN436008), tomato yellow spot alphasatellite (KX348228), and cleome leaf crumple alphasatellite (FN436007), all belonging to the *Clecrusatellite* genus (Figure 4a).

### 3.3. Recombination Analyses

Seven methods implemented in the RPD4 recombination analysis program supported that ToYSV had a recombination origin and all ToYSV sequences, both sequences reported in this work and those previously reported in GenBank, shared the same recombination event (Table 4). The recombinant breakpoint for ToYSV sequences was located in the Rep region and involved a ToDfLV (Sample 589) as a major parent, and a Brazilian isolate of sida yellow mosaic virus (SiYMV; AY090558) as a minor parent. The recombination analysis revealed that PepBLV was involved in recombination events as a mayor parent of SoMBoV (HM585435) and CeYSV (JN419002) (Table 4).

Moreover, inter-species recombination events were detected for SbBMV and ToYSV (Table 4, Figure S2). The split-decomposition networks from 10 SbBMV aligned sequences showed several conflicting phylogenetic signals, possibly due to recombination (Figure 2b). When pepper samples 145, 317, and EF01648 sequences were removed from the data set, the conflicting signals disappeared in the phylogenetic network, and the PHI test analysis (*p* = 0.556) indicated the absence of recombination (Figure 2c). Both methods, RPD4 and SplitsTree4, identified the same recombinant SbBMV isolates for DNA-A (Table 4), whereas for DNA-B, the PHI test did not indicate evidence of recombination (*p* = 0.785), and RDP4 detected sample 263 as recombinant between the isolates 654 and 271 (Table 4).

On the other hand, a multiple-reticulated network was obtained for 30 ToYSV DNA-A aligned sequences with PHI test (*p* < 0.001), suggesting intra-species recombination for ToYSV (Figure S1b). RPD4 detected intra-species recombination only for the DNA-B of the same isolates (Table 4). 

## 4. Discussion

The importance of begomovirus as causative agents of pepper diseases in NWA was confirmed since the detection of 51.48% of the samples were infected with these viruses. In Brazil, yield losses between 28 and 45% have been attributed to begomovirus infection in pepper, indicating the potential importance of such diseases [55]. The diversity of begomoviruses infecting pepper has been determined in this study using the RCA-NGS approach for the first time in Argentina. Six pepper-associated begomovirus species were identified in this study: SbBMV, ToYSV, ToYVSV, ToDfLV, SiGMBRV, and PepBLV, a proposed new species.

SbBMV was the most abundant begomovirus detected, whereas, in Brazil, ToSRV was found to be the most important and widespread begomovirus in pepper [55]. Since the selection of the samples was based on the observed diversity of RCA-RFLP patterns, the higher abundance of SbBMV could also be related to its higher diversity. SbBMV was previously reported in soybean crops in Argentina [56]. Here, the DNA-B sequence was reported for the first time, thereby completing the genome characterization of SbBMV. We detected three possible SbBMV recombinants (pepper samples 145 and 317, and soybean isolate EF016486) between isolates of SbBMV using RDP4 and SplitsTree4 (Table 4, Figure 2b). DNA-A of SbMBV showed higher variability in NWA, only involving two hosts (pepper and soybean) in a shared geographical area. The detected intra-species recombination events could also contribute to this variability. Moreover, intra-species recombination was detected for ToYSV (Table 4). Inter-species recombination is an important process during the evolution of begomoviruses and has a role in the emergence of a new strain [57]. ToYSV was the second most abundant species detected infecting pepper and was previously identified in bean and chia in Argentina [56,58]. ToYSV has a wide host range, including *Solanaceae*, *Amaranthaceae*, *Fabaceae*, and *Lamiaceae* families [59,60,61]. ToYSV is more closely related to sida-infecting begomovirus, and previous analysis of recombination indicated that a fragment corresponding to capsid protein in ToYSV is probably derived from sida mottle virus (SiMoV; [62]). In this study, the recombination event for ToYSV was detected in the replication-associated protein -AC4-intergenic region fragment and involved a ToDfLV as the major parent and SiYMV as the minor parent (Table 4, Figure S2).

We also identified ToDfLV, ToYVSV, and SiGMBRV infecting pepper. ToDfLV was previously reported infecting tomato in Argentina [63]. ToYVSV was first identified infecting potato in Argentina and Brazil; then, it was reported infecting tomato in Brazil, Argentina, Uruguay, and Chile, and bean in Argentina [64,65,66,67]. ToYSV was also reported infecting pepper, but with minor importance, in Brazil [16,55]. Consequently, ToYVSV is one of the species with the greatest distribution among the bordering countries. SiGMBRV was reported infecting Sida sp. in Brazil and bean in Argentina [68,69]. Briefly, of the six begomovirus species detected in pepper in Argentina, only ToYVSV was also detected infecting pepper in Brazil, and ToYSV was reported as being able to infect biolistically inoculated peppers [59].

These results confirm that RCA-NGS is a powerful tool for viral DNA diversity exploration and is challenging the way that we identify and classify viruses [70]. We were able to reconstruct the complete genome for both components in most samples, even when the proportion of pepper reads in the RCA amplified DNA was high. As mentioned earlier, a new species denominated PepBLV was identified and characterized. Our results showed that the complete DNA-A sequence of PepBLV generated by RCA-NGS exhibited 99.84% nucleotide identity with the Sanger-sequenced one, supporting the strategy of RCA-NGS de novo assembly of the full-length genome used here. We also compared the RFLP patterns obtained from RCA-RFLP and in silico RFLPs of de novo assembled sequences for each sample to verify begomovirus identification (Table S1). Although we used four different restriction endonuclease enzymes that recognized 6-bp sequences for the RCA-RFLP analysis, we suggested choosing one with 4-bp recognized sequences. A step of RCA-RFLP could be a good procedure for screening the samples for sequencing and for validating the results obtained by RCA-NGS. We used these RCA-RFLP comparisons to assess the potential problem of generating chimeric begomovirus genomes that could derive from the assembly of sequences belonging to different species in a mixed infection sample. There are several reports about the occurrence of mixed infection in the field, caused by the transmission of the viruses by the same vector [12,55,63,71]. However, in this work, we only detected single infections in pepper.

In addition, we identified a new alphasatellite species, which we denominated tomato yellow spot alphasatellite 2 (ToYSA 2), associated with ToYSV in pepper and *L. japonicus.* This was the first record of an alphasatellite infecting pepper and the first alphasatellite reported in Argentina. ToYSA 2 is phylogenetically related to alphasatellites associated with new world begomoviruses in non-cultivated plants, like euphorbia yellow mosaic alphasatellite (EuYMA) identified in *Euphorbia heterophylla* and *Sida* spp., cleome leaf crumple alphasatellite (ClLCrA) in *Cleome affinis,* and tomato yellow spot alphasatellite (ToYSA) in *Leonurus sibiricus* (synonym *L. japonicus)* [68,71,72]. We found ToYSA 2 associated with ToYSV in *L. japonicus* and pepper, showing that it could be transferred from weed to crop or vice versa. ToYSV was previously identified infecting *L. japonicus* in Brazil and Paraguay; those findings, along with this new identification in Argentina, reinforce its role as a potential source of inoculum to tomato, soybean, bean, and pepper crops [60,61]. EuYMA is capable of symptom modulation, viral accumulation, and whitefly transmission of euphorbia yellow mosaic virus (EuYMV), thereby potentially interfering with virus dissemination in the field [73].

Finally, this work was a clear example that the combination of an ecogenomic approach with powerful tools like RCA-NGS to reconstruct whole-genome DNA viruses is fundamental in ecological and evolutionary genomic studies.

## Figures and Tables

**Table 1:** Details of samples, virus isolates (country, province, location, host species-sample number and year of collection), Illumina HiSeq 1500 system data generated (number of raw paired reads, number of pairs after quality trimming, percentage of the sample that mapped against *C. annum*), and results of the de novo assembly contig obtained by IVA (Iterative Virus Assembler).

Sample	Isolate	Raw Paired Reads	Reads Post Trimming	% *C. annum*	Viral Contigs
145	AR:Salta:Pichanal:Pepper145:2005	4,612,436	2,469,680	46.64	2
169	AR:Salta:Pichanal:Pepper169:2006	10,002,748	4,604,741	66.78	2
262	AR:Salta:Pichanal:Pepper262:2007	8,874,712	4,060,381	76.03	2
263	AR:Salta:Pichanal:Pepper263:2007	10,935,998	4,851,060	44.78	2
265	AR:Salta:Pichanal:Pepper265:2007	4,846,478	2,218,946	32.66	2
267	AR:Salta:Pichanal:Pepper267:2007	10,927,593	4,694,431	54.31	2
269	AR:Salta:Pichanal:Pepper269:2007	9,735,179	4,060,166	77.69	2
271	AR:Salta:Pichanal:Pepper271:2007	10,869,197	4,959,285	54.16	2
302	AR:Salta:Orán: Pepper302:2007	5,122,034	2,563,396	78.54	1
309	AR:Salta:Pichanal:Pepper309:2007	9,401,380	4,537,658	85.43	2
316	AR:Salta:Pichanal:Pepper316:2008	9,626,908	5,106,324	25.19	2
317	AR:Salta:Pichanal:Pepper317:2008	3,952,259	1,675,427	32.37	2
322	AR:Salta:Pichanal:Pepper322:2008	10,178,983	4,918,919	12.87	2
328	AR:Jujuy:Yuto: Pepper328:2008	5,322,366	2,446,806	21.44	3
423	AR:Jujuy:Yuto: Pepper423:2008	4,965,540	2,438,148	87.37	0
588	AR:Jujuy:Yuto: Pepper588:2011	3,955,481	1,903,671	59.05	2
589	AR:Jujuy:Yuto: Pepper589:2011	10,554,833	4,753,535	59.32	2
654	AR:Salta:Orán: Pepper654:2014	10,858,704	4,626,688	75.22	1
658	AR:Salta:Orán: Pepper658:2014	4,316,078	2,066,084	86.22	2
663	AR:Salta:Orán: Pepper663:2014	10,342,387	4,946,984	83.71	2

**Table 2:** Details of begomovirus and alphasatellite sequences generated from rolling circle amplification-next generation sequencing (RCA-NGS) data.

Sample	Assembly Method	Coverage	Genome Length	Genomic Component	Virus Acronym	GenBank Accession Number	SDT % Identity	Best BLAST Hit
145	de novo	7831.28	2603	DNA-A	SbBMV	MN486865	95.4	Soybean blistering mosaic virus DNA-A (EF016486)
		7838.68	2551	DNA-B	SbBMV	MN486866	82.8	Solanum mosaic Bolivia virus DNA-B (HM585436)
169	de novo	7836.68	2586	DNA-A	SbBMV	MN486867	94	Soybean blistering mosaic virus DNA-A (EF016486)
		7848.72	2589	DNA-B	SbBMV	MN486868	58.7	Solanum mosaic Bolivia virus DNA-B (HM585436)
262	de novo	7437.22	2466	DNA-A	ToYSV	MN508241	98.2	Tomato yellow spot virus DNA-A (KJ742419)
		7742.25	2602	DNA-B	ToYSV	MN508242	95.5	Tomato yellow spot virus DNA-B (KJ742420)
263	de novo	7859.79	2603	DNA-A	SbBMV	MN486869	93.4	Soybean blistering mosaic virus DNA-A (EF016486)
		7855.25	2551	DNA-B	SbBMV	MN486870	83	Solanum mosaic Bolivia virus DNA-B (HM585436)
265	de novo	7803.97	2603	DNA-A	SbBMV	MN486871	93.4	Soybean blistering mosaic virus DNA-A (EF016486)
		7855.33	2552	DNA-B	SbBMV	MN486872	82.8	Solanum mosaic Bolivia virus DNA-B (HM585436)
267	de novo	7790.12	2632	DNA-A	ToYSV	MN508243	97.6	Tomato yellow spot virus DNA-A (KJ742419)
		7836.16	2594	DNA-B	ToYSV	MN508244	96	Tomato yellow spot virus DNA-B (KJ742420)
269	de novo	6504.38	2632	DNA-A	ToYSV	MN508245	97.6	Tomato yellow spot virus DNA-A (KJ742419)
		7627.65	2594	DNA-B	ToYSV	MN508246	97.6	Tomato yellow spot virus DNA-B (KJ742420)
271	de novo	7809.05	2592	DNA-A	SbBMV	MN508206	92.6	Soybean blistering mosaic virus DNA-A (EF016486)
		7859.85	2578	DNA-B	SbBMV	MN508207	83.1	Solanum mosaic Bolivia virus DNA-B (HM585436)
302	filtering + reference	307.31	2653	DNA-A	SiGMBRV	MN508222	95.4	Sida Brazil virus DNA A (KY555798)
		221.93	2399	DNA-B	SiGMBRV	MN508223	94.9	Sida Brazil virus DNA B (KY555799)
309	de novo	2396.62	2564	DNA-A	ToYVSV	MN508216	98.7	Tomato yellow vein streak virus DNA-A (KC136337)
		5370.95	2560	DNA-B	ToYVSV	MN508217	95.8	Tomato yellow vein streak virus DNA-B (KC136338)
316	de novo	7863.88	2603	DNA-A	SbBMV	MN508208	93.4	Soybean blistering mosaic virus DNA-A (EF016486)
		7882.99	2549	DNA-B	SbBMV	MN508209	82.8	Solanum mosaic Bolivia virus DNA-B (HM585436)
317	de novo	7803.6	2603	DNA-A	SbBMV	MN508210	95.5	Soybean blistering mosaic virus DNA-A (EF016486)
		7833.51	2584	DNA-B	SbBMV	MN508211	58.3	Solanum mosaic Bolivia virus DNA-B (HM585436)
322	de novo	7865.51	2605	DNA-A	SbBMV	MN508212	94	Soybean blistering mosaic virus DNA-A (EF016486)
		7885.42	2550	DNA-B	SbBMV	MN508213	82.9	Solanum mosaic Bolivia virus DNA-B (HM585436)
328	de novo	7787.35	2641	DNA-A	ToYSV	MN508247	95.8	Tomato yellow spot virus DNA-A (KJ742419)
		7846.58	2609	DNA-B	ToYSV	MN508248	94.5	Tomato yellow spot virus DNA-B (KJ742420)
423	filtering + de novo	938.35	1350	Alphasastellite	ToYSA 2	MN518745	85.1	Tomato yellow spot alphasatellite (KX348228)
		432.04	2594	DNA-B	ToYSV	MN518746	95.9	Tomato yellow spot virus DNA-B (KJ742420)
588	filtering + de novo	7253.73	2540	DNA-A	ToDfLV	MN508218	97.4	Tomato dwarf leaf virus DNA-A (JN564749)
		7777.29	2501	DNA-B	ToDfLV	MN508219	97.2	Tomato dwarf leaf virus DNA-B (JN564750)
589	de novo	7834.03	2539	DNA-A	ToDfLV	MN508220	98.1	Tomato dwarf leaf virus DNA-A (JN564749)
		7818.73	2501	DNA-B	ToDfLV	MN508221	99.2	Tomato dwarf leaf virus DNA-B (JN564750)
654	filtering + reference	7767.16	2603	DNA-A	SbBMV	MN508214	93.4	Soybean blistering mosaic virus DNA-A (EF016486)
		7808	2641	DNA-B	SbBMV	MN508215	82.9	Solanum mosaic Bolivia virus DNA-B (HM585436)
658	de novo	3845.53	2630	DNA-A	ToYSV	MN518733	98.5	Tomato yellow spot virus DNA-A (FJ538207)
		5562.73	2591	DNA-B	ToYSV	MN518734	98.5	Tomato yellow spot virus DNA-B (KJ742420)
663	filtering + de novo	4660.23	2651	DNA-A	PepBLV	MN518735	83.1	Solanum mosaic Bolivia virus DNA-A (HM585435)
		1256.06	2614	DNA-B	PepBLV	MN518736	83.7	Sida mosaic Bolivia virus DNA-B (HM585442)

**Table 3:** Details of begomovirus and alphasatellite sequences generated by cloning and Sanger sequencing.

Sample	Isolate (Country: Province: Location: Host Species-Sample Number: Year of Collection)	GenBank Accession Number	Viral Sequence Length	Virus Acronym/Genomic Component	SDT % Identity of BLAST Hit/Acronym of Begomovirus Identified /GenBank Accession Number
417	AR: Jujuy:Yuto: Leonurus417:2014	MN518741	2632	ToYSV/DNA-A	98.9% ToYSV DNA-A (KJ742419)
		MN518742	2592	ToYSV/DNA-B	96% ToYSV DNA-B (KJ742420)
		MN518743	1350	ToYSA 2	84.2% Tomato yellow spot alphasatellite (KX348228)
		MN518744	1350	ToYSA 2	84.2% Tomato yellow spot alphasatellite (KX348228)
663	AR:Salta:Orán: Pepper663:2014	MN518737 MN518738	2651	PepBLV/DNA-A	83% SoMBoV DNA-A (HM585435)
		MN518739 MN518740	2614	PepBLV/DNA-B	78% SiMBV1 DNA-B (NC_015044)

**Table 4:** Recombination events detected for begomovirus infecting pepper in northwestern Argentina (NWA). Results based on data set comprising all begomoviruses from South America and analyzed using RDP4 (Recombination Detection Program).

Recombinant Sequence	Major Parental	Minor Parental	Methods	Av. *p*-Value	Beginning Breakpoint	Ending Breakpoint
DNA-A						
ToYSV (DQ336350, Sample 658, 328, 269, 267, 262, NC007726, KX348179, KX348178, KX348177, KX348176, KX348175, KX348174, KX348173, KX348172, KX348171, KX348170, KX348169, KX348168, KX348167, KX348166, KX348165, (KJ742419, KC706628, JX513952, FJ538207)	ToDfLV (Sample 589)	SiYMV (AY090558)	RGBMCST	1.057 × 10^−12^	2664	2029
SoMBoV (HM585435)	PepBLV (Sample 663)	SbBMV (EF016486)	RBMCST	2.432 × 10^−18^	30	1948
SbBMV (Sample 317, 145)	SbBMV (Sample 322)	SbBMV (Sample 271)	RGBMCST	5.130 × 10^−17^	201	584
CeYSV (JN419002)	PepBLV (Sample 663)	CleGMV (NC_015397)	RGBMCST	2.312 × 10^−9^	2001	2222
SbBMV (EF016486)	SbBMV (Sample 322, 654, 316, 265, 263, 169)	SbBMV (Sample 271)	RGBMCST	3.971 × 10^−11^	867	176
DNA-B						
ToYSV (KX348212)	ToYSV (KX34820)	ToYSV (KX348216)	RMCST	9.303 × 10^−17^	58	1373
ToYSV (KX348211)	ToYSV (KX348213)	ToYSV (KX348222)	RGBMCST	3.369 × 10^−7^	437	1257
ToYSV (KX348205)	ToYSV (KX348223)	ToYSV (KX348206)	RGMCST	7.057 × 10^−7^	982	2510
ToYSV (Sample 269)	ToYSV (KJ742420)	ToYSV (Sample 328)	RGBMCST	1.856 × 10^−6^	2203	2406
SbBMV (Sample 263)	SbBMV (Sample 654)	SbBMV (Sample 271)	RGBMCST	5.872 × 10^−7^	2256	2336

## Supplementary Materials

The following are available online at https://www.mdpi.com/1999-4915/12/2/202/s1, Figure S1: (a) Pairwise identity matrix of all ToYSV DNA-A sequences inferred using SDT v1.2; (b) Phylogenetic networks for ToYSV DNA-A sequences obtained with NNet algorithm using Splits Tree and significant PHI test. Figure S2: Schematic representation of recombination events detected in DNA-A and DNA-B sequences using RDP4. ORFs in relation to the recombination breakpoints are indicated with arrows above recombinants. Sequence fragments were coloured according to their associated species. (a) Intra-species recombination events; (b) Inter-species recombination events. Table S1: RFLPs patterns (pb) corresponding to each pepper-sample generated by RCA-RFLP and *in silic*o-RFLP from assembled DNA-A and DNA-B begomovirus sequences. * sequence of alphasatellite from sample 423. Bands corresponding to DNA-A are in bold.
