# Supplementary material for: Reconstruction and Characterization of Full-Length Begomovirus and Alphasatellite Genomes Infecting Pepper through Metagenomics
Source: Viruses. 2020 Feb 11;12(2):202. doi: 10.3390/v12020202 (PMC7077291; doi:10.3390/v12020202)
Supplement: Supplementary file 1 [file viruses-12-00202-s001.pdf]

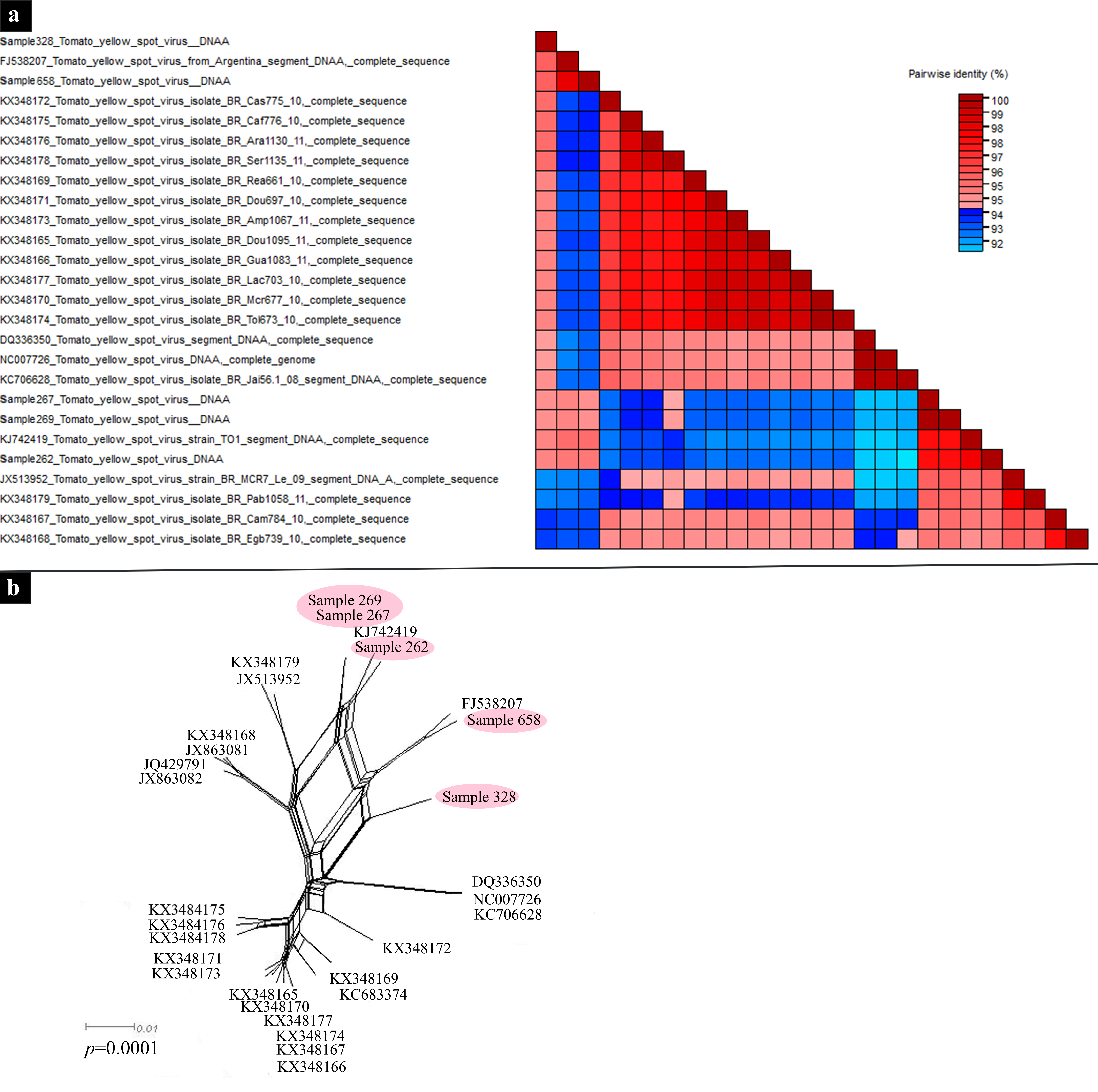
a
Sample328_Tomato_yellow_spot_virus__DNAA
FJ538207_Tomato_yellow_spot_virus_from_Argentina_segment_DNAA,_complete_sequence
Sample658_Tomato_yellow_spot_virus__DNAA
KX348172_Tomato_yellow_spot_virus_isolate_BR_Cas775_10,_complete_sequence
KX348175_Tomato_yellow_spot_virus_isolate_BR_Caf776_10,_complete_sequence
KX348176_Tomato_yellow_spot_virus_isolate_BR_Ara1130_11,_complete_sequence
KX348178_Tomato_yellow_spot_virus_isolate_BR_Ser1135_11,_complete_sequence
KX348169_Tomato_yellow_spot_virus_isolate_BR_Rea661_10,_complete_sequence
KX348171_Tomato_yellow_spot_virus_isolate_BR_Dou697_10,_complete_sequence
KX348173_Tomato_yellow_spot_virus_isolate_BR_Amp1067_11,_complete_sequence
KX348165_Tomato_yellow_spot_virus_isolate_BR_Dou1095_11,_complete_sequence
KX348166_Tomato_yellow_spot_virus_isolate_BR_Gua1083_11,_complete_sequence
KX348177_Tomato_yellow_spot_virus_isolate_BR_Lac703_10,_complete_sequence
KX348170_Tomato_yellow_spot_virus_isolate_BR_Mcr677_10,_complete_sequence
KX348174_Tomato_yellow_spot_virus_isolate_BR_Tol673_10,_complete_sequence
DQ336350_Tomato_yellow_spot_virus_segment_DNAA,_complete_sequence
NC007726_Tomato_yellow_spot_virus_DNAA,_complete_genome
KC706628_Tomato_yellow_spot_virus_isolate_BR_Jai56.1_08_segment_DNAA,_complete_sequence
Sample267_Tomato_yellow_spot_virus__DNAA
Sample269_Tomato_yellow_spot_virus__DNAA
KJ742419_Tomato_yellow_spot_virus_strain_TO1_segment_DNAA,_complete_sequence
Sample262_Tomato_yellow_spot_virus_DNAA
JX513952_Tomato_yellow_spot_virus_strain_BR_MCR7_Le_09_segment_DNA_A,_complete_sequence
KX348179_Tomato_yellow_spot_virus_isolate_BR_Pab1058_11,_complete_sequence
KX348167_Tomato_yellow_spot_virus_isolate_BR_Cam784_10,_complete_sequence
KX348168_Tomato_yellow_spot_virus_isolate_BR_Egb739_10,_complete_sequence
Pairwise identity (%)
100
99
98
98
97
96
95
95
94
93
92
b
Sample 269
Sample 267
KJ742419
Sample 262
KX348179
JX513952
FJ538207
Sample 658
KX348168
JX863081
JQ429791
JX863082
Sample 328
DQ336350
NC007726
KC706628
KX3484175
KX3484176
KX3484178
KX348171
KX348173
KX348172
KX348169
KC683374
KX348165
KX348170
KX348177
KX348174
KX348167
KX348166
0.01
p=0.0001
